# Tautomerism in Azo and Azomethyne Dyes: When and If Theory Meets Experiment

**DOI:** 10.3390/molecules24122252

**Published:** 2019-06-17

**Authors:** Liudmil Antonov

**Affiliations:** Institute of Organic Chemistry with Centre of Phytochemistry, Bulgarian Academy of Sciences, Sofia 1113, Bulgaria; lantonov@orgchm.bas.bg

**Keywords:** DFT, tautomerism, azonaphthols, Schiff bases, optical spectroscopy, chemometrics

## Abstract

The performance of 26 hybrid density functionals was tested against a tautomeric dataset (TautData), containing experimental information for the keto-enol tautomeric equilibrium in 16 tautomeric azodyes and Schiff bases in cyclohexane, carbon tetrachloride and acetonitrile. The results have shown that MN12-SX, BHandH and M06-2X can be used to describe the tautomeric state of the core structures in the frame of ~0.5 kcal/mol error and correctly predict the tautomeric state in respect of dominating tautomeric form. Among them MN12-SX is the best performer, although it fails to describe the nonplanarity of some of the enol tautomers. The same experimental dataset was used to develop and test a special DFT functional (TautLYP) aimed at describing the tautomeric state in azo- and azomethyne compounds in solution when nonspecific solvents are used.

## 1. Introduction

Prototropic tautomerism [1,2,3,4,5,6,7,8,9,10,11,12] is one of the most important phenomena in organic chemistry despite the relatively small proportion of molecules in which it can occur. Tautomers are the chameleons of chemistry, capable of changing by a simple change of environment from an apparently established structure to another, then back again when the original conditions are restored. The importance of the tautomerism and proton transfer [12] in the life science, drug design and technology makes it vital to know and/or to predict which tautomer is the major one, since not only the structure, but chemical properties are bound up with this. 

**Scheme 1:**
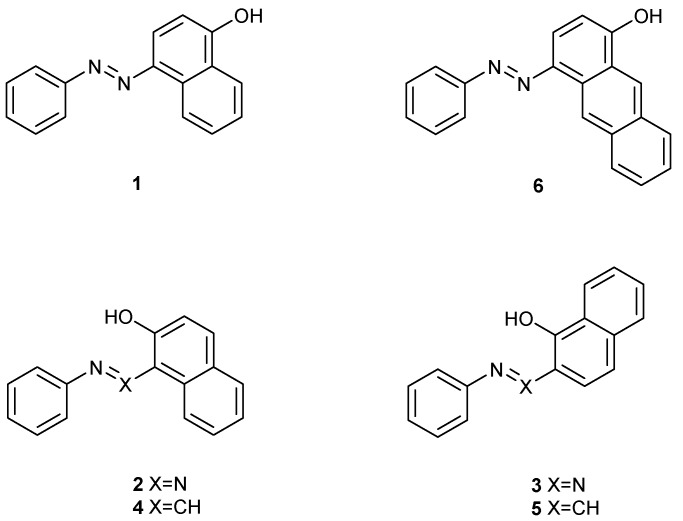
Core tautomeric dyes used in this study.

According to the classification of dyes, more than half of them are azo compounds and a substantial part are tautomeric or potentially tautomeric [13,14,15,16]. Compound **1** (Scheme 1), one of the first tautomeric compounds discovered [17] and studied almost one and half century [18], is a typical example. Experimental and theoretical investigations of the tautomerism have a solid practical reason, because the color and the stability of the azodyes are strongly influenced by their tautomerism [19]. The Schiff bases are another important class of dyes exhibiting interesting photo- and thermochromic properties and bioactivity, again, in most of the cases, related to their tautomerism [20,21,22].

**Scheme 2:**
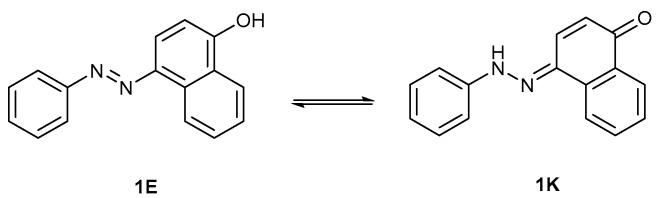
Tautomeric equilibrium in **1** given as an example for the structures from Scheme 1.

While the potentially tautomeric structures can be easily distinguished either theoretically or experimentally [23], the study of real tautomeric equilibria in solution is a challenge. On one side, the real equilibrium from the viewpoint of the detection limits of analytical chemistry means Gibb’s free energy in the range roughly from −2 to +2 kcal/mol, which is a challenge for quantum chemistry even now [24]. On the other side, the exact estimation of the ΔG values (derived from the tautomeric constant K_T_, defined as keto/enol, [**K**]/[**E**] ratio, Scheme 2), requires isolated tautomeric forms, a condition that is impossible in most of the cases. A major breakthrough in the UV-Vis spectroscopy was the development of chemometric methods to obtain the absorption spectra of the individual tautomers, even though these are never present in their pure form, and then to estimate the tautomeric constants as function of a number of external parameters (temperature, irradiation, solvents, pH, and concentration) [25,26]. The availability of such experimental data has given us the opportunity to check the reliability of the quantum-chemical methods in predicting the position of the tautomeric equilibria in selected azonaphthols and related Schiff bases in solution [24,27]. The results for these dyes and for the tautomeric compounds in general, clearly indicate that there is no universal reliable theoretical solution and the predicted tautomeric shift, often wrong, depends strongly on the theoretical background.

The affordable accuracy of quantum chemistry in respect of correct prediction of tautomeric equilibria depends on one side on the molecular size and particularly on the total number of atoms, and on the other, on the requirement to take into account the effect of the solvent. Currently molecules with up to 10 atoms can be very accurately studied by coupled cluster theory, similar to 100 atoms with second order Moller-Plesset perturbation theory (MP2), similar to 1000 atoms with DFT and beyond this number with semiempirical quantum chemistry and force-field methods [28]. The previous results obtained by using MP2 for tautomeric azonaphthols have shown that this level of theory very strongly overestimates the enol tautomer irrespective of the used basis set [27]. This along with the computational affordability directs the possible solution into the DFT field. There is a substantial number of datasets used for benchmarking and/or density functional development in the field of thermochemistry, kinetics, and noncovalent interactions [29,30,31,32,33]. To our best knowledge, no attempts have been made to develop a density functional that describes reasonably well the relative energies of substantial set of tautomeric compounds. The obvious reason is that there is no way for systematic improvement of the DFT functionals performance, especially in the absence of experimental data to test the theoretical development.

**Scheme 3:**
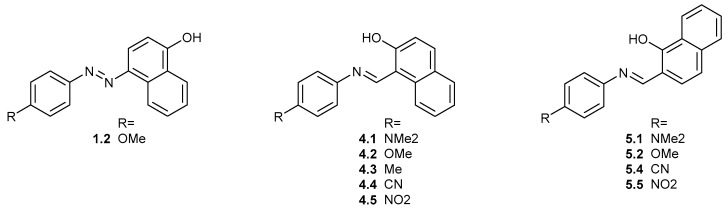
Substituted compounds derived from **1**, **4** and **5**.

Therefore, the aim of the current communication is to introduce a tautomeric dataset (TautData), containing reliable and precise experimental data for the tautomeric equilibrium of basic dye structures in solution (Scheme 1 and Scheme 3) and to test how the most promising density functionals, MP2 and coupled cluster methods predict the tautomerism of these compounds. In addition, the available experimental information has been used to develop a cheap solution for correct description of the tautomerism of the dyes listed in Scheme 1. The reliability of the new tautomeric functional (TautLYP) is compared with the others in a large collection of dyes derived from the structures in Scheme 1 (Scheme 3).

## 2. Results and Discussion

The compounds used for benchmarking and DFT functional development (Scheme 1) represent core structures for azo- and corresponding azomethyne dyes [15,34]. Compound **1** is one of the first tautomeric dyes discovered. Compounds **2** and **3** are the core structures of the major class (>60%) of the industrial azodyes (neutral and their metal complexes). Compounds **4** and **5** exhibit interesting photochemical and thermochemical properties in solution and solid state [20,35]. Their tautomeric behavior in solution is very different. In **2**–**5** the existing intramolecular hydrogen bonding limits the effect of the solvent on the shift of the tautomeric equilibrium. As a result, the tautomeric constants can be changed by change of the solvent in a relatively narrow range. The specific effect of the solvent is also different; the strength of the intramolecular hydrogen bonding in **4** and **5** limits the effect of the proton acceptor solvents, while the same bond in **2** and **3** is weak and can be easily broken [36,37]. The proton acceptor/donor sites in the tautomers of **1** are accessible for the solvents, which allows for a large scale of the tautomeric constant change [38]. In **6,** due to aromatic reasons, the tautomeric equilibrium is shifted almost fully to the keto form [13,39,40]. The structural effects are also different; substitution on para position of the phenyl ring in the azodyes leads to a shift of the tautomeric equilibrium towards the enol form when electron donor group is implemented and vice versa. The effect of the substitution in the Schiff bases is limited due to the substantial nonplanarity of the phenyl ring in respect of the overall conjugated system [41].

Keeping in mind that as a rule the solvent effect on the tautomeric equilibrium is specific, experimental data obtained in cyclohexane are used for the benchmarking in the current study. In this way it is assumed that the implicit solvation describes the effect of the solvent. The corresponding ΔG values at room temperature for compounds **1**–**6** are listed in the last row of Table 1. The positive values indicate a more stable enol form and vice versa. It is clearly seen that the set of **1**–**6** covers the whole range of changes, from 1.42 in **4** to −1.31 in **6**, which along with the variety of structural features discussed above, makes the selection of dyes very suitable for benchmarking. The experimental data are compared in Table 1 to the predicted relative stabilities of the tautomers generated by variety of hybrid GGA and meta-GGA density functionals. Their order of appearance depends on the statistical data of their performance presented in Table 2. The selection of the functionals is related to their popularity, use in investigation of tautomeric systems and previous benchmarking of **1** [18] and **1**–**3** [27].

It seems from the data that no one of the existing density functionals fits the experiment perfectly. Linearity, shown in Table 1, can be used for monitoring whether the general tendency of predicting is correct or not. Basically, the perfect situation should have correlation and slope tending to 1 and a zero intercept. Some of the functionals give highly correlated results (M06-2X, BHandHLYP, ωB97X), while in other cases the predictions scatter (M06-HF, TPSS, O3LYP). The description the tautomeric state of the studied compounds is quite unrealistic in the case of HF and MP2. In most of the cases the slope tends to 1, which with the relatively good correlations indicates that corresponding functional correctly describes the relative structural aspects of the tautomerism (SOGGA11-X for instance). The intercept roughly shows if the theory overestimates one or another tautomer in the tautomeric mixture; most of the functionals overestimate the stability (negative slope) of the keto tautomer, some of them very strongly (CAM-B3LYP and below). The statistical data in Table 2 follows the major conclusions up to now. Generally it is very difficult to write a clear conclusion about tendencies; the best behaving density functionals belong to different classes (GH/RSH mGGA/GGA), so there is no clear relation with the HF exchange and even functionals belonging to the same family (BHandH, BHandHLYP, B3LYP) behave in a different way. It is obviously that MN12-SX, BHandH and M06-2X can be used to describe the tautomeric state of **1**–**6** in the frame of ~0.5 kcal/mol error and correctly predict the tautomeric state in respect of dominating tautomeric form (i.e. positive/negative ΔE). In the frame of 1 kcal/mol precision SOGGA11-X, M-11 and BHandHLYP could be also used. However, this is a general statistical conclusion which should be taken with care. If BHandHLYP is considered one example, it predicts molar fraction of the keto tautomer as 33, 75, 90, 29, 61, 99 (in %, calculated from the corresponding ΔE values in Table 1) for **1**–**6** respectively. At the same time the experimental values are 9, 33, 60, 8, 17, 90, which means that the position of the tautomeric equilibrium in **2** and **5** is predicted completely wrong, while the values of **1**, **3** and **4** strongly underestimate the contribution of the enol tautomer.

It is obvious from Table 1 and Table 2 that TautLYP describes the experimental dataset much better compared to the next best performers, which is not surprising since it is fitted against exactly this experiment. Therefore, it is needed to check it and the others’ reliability against different datasets. For this purpose, structural data (bond length and dihedral angles) of **1** and **6** will be used along with equilibrium data for the dyes from Scheme 3.

Currently, structural data for the enol and keto tautomers of **1** are available in the literature [18] along with data for the fixed enol tautomer (OMe compound) of **6** [69]. In the case of compounds **2**–**5** even if crystallographic data are available the structures contain contribution of both tautomers [70,71], which makes the structural information unsuitable for benchmarking.

The structural data along with the predicted results for the density functionals from TautLYP to BHandHLYP are collected in Table S1, Supplementary information. In Table 3 the statistical evaluation of the performance of the functionals is given.

The results indicate that all studied DFT functionals describe the bond lengths reasonably well. The differences are very slight (except in the case of BHandH) to speculate about best performers. In respect of the angles description two distinguished groups are seen, depending on the non-planarity description. MN12-SX, BHandH and M11 predict the enol form planar, which reflects in substantially higher MAE and MAPE. In respect of the total MAPE TautLYP and M06-2X are the best performers. 

Experimental data in CCl_4_ and acetonitrile for the tautomeric state of the core structures and their substituted compounds are collected in Table 4. As in the case of cyclohexane, the solvents have been selected to avoid specific solute-solvent interactions and to assume implicit solvation model. The performance of the best functionals from Table 2 is compared with that of B3LYP in Table 5. The statistical results are based on the data from Table S2. It is seen that TautLYP predicts the experiment slightly better comparing to MN12-SX, but the information from Table 3 should also be taken into account for the final decision which of them to use. Comparing to B3LYP both have MAPE in orders better.

Availability of CCSD(T) data in the gas phase makes possible to compare the results with these of TautLYP. Of course, there is a difference in the linear equations of TautLYP in cyclohexane (Table 1) and in the gas phase (Table 6) in respect of the offset. The gas phase equation gives systematic predominance of the enol tautomers, which is reasonable because the higher dipole moment of the keto forms, giving difference in the implicit solvation model in cyclohexane, is not accounted. Keeping in mind that for the CCSD(T) calculations MP2 optimized geometry is used, in Table 6 the results for TautLYP and TautLYP//MP2 are presented. Although the linearity parameters are almost the same there is difference in the prediction of the ΔE values for the azo compounds in the frame of ~0.2 kcal/mol, which is a result from the increased twisting of the phenyl ring in the MP2 optimization geometry. In the case of **4** and **5** where the angles predicted by TautLYP and MP2 are near, the difference in the ΔE values is small. Comparing MP3//MP2 and MP4//MP2 with TautLYP, it is seen that the former strongly overestimates the keto tautomer, while the latter has relatively good prediction ability. The overall impression for CCSD(T) is that the enol form is overestimated, especially in the case of **6**. A comparison of the CCSD(T) and TautLYP//MP2 predicted ΔE values shows that they are almost identical for **1**, strongly different in **6** and well correlated in the case of **2**–**5** (with a difference of 0.9–1.1 kcal/mol in respect of the enol stabilization by CCSD(T)).

Further studies of the performance of TautLYP by using the standard databases for DFT development, as well as comparison with CCSD(T) calculations in solution, could shed light on the applicability of this density functional for general purposes and for predicting the equilibrium state of other tautomeric systems in solution. The calculations are in progress and the results will be reported later.

## 3. Materials and Methods

In total, 35 compounds (**1**–**6** and derived from them) were synthesized, purified and characterized according to the standard procedures [14,15]. For each of them the protocol for determination of the tautomeric constants as described previously was applied [26,72]. The protocol includes UV-Vis spectra measurements followed by a chemometric data processing [72]. To make the data comparable, all spectra were measured at the same instrumental conditions (scan speed, detector output and spectral slit) on Jasco V570 UV-Vis-NIR double beam spectrometer (JASCO International Co. Ltd., Tokyo, Japan) equipped with a thermostatic cell holder (Huber MPC-K6 thermostat with 1 °C precision (Peter Huber Kältemaschinenbau AG, Offenburg, Germany)) in spectral grade solvents (cyclohexane and acetonitrile, both Multisolvent grade from Sharlau (Scharlab, Barselona, Spain)) at 25 °C. The obtained spectra of each compound were processed using resolution of overlapping bands in order to get the molar fractions of the keto and enol tautomers and their spectral characteristics in the corresponding solvent. The chemometric protocol is described in detail, together with examples in [72]. The tautomeric molar fractions allow for calculation of the tautomeric constant K_T_ (defined as [**K**]/[**E**]) and hence, evaluation of the corresponding ΔG value, which is finally included in TautData.

For the aims of benchmarking, in the TautData dataset are finally included only precise ΔG values in cyclohexane and acetonitrile, obtained based on molar fractions of the enol tautomer in the frame ±5%. The data for CCl_4_ are taken from the literature and are obtained previous by us using the same protocol [36,41,73,74]. Keeping in mind that they are obtained at different time, the precision might be lower comparing to these in cyclohexane and acetonitrile due to slightly different experimental conditions.

The quantum chemical calculations in current study were performed by using Gaussian 09 Rev. D.01 program suite [75]. In all cases 6-31++G** basis set was applied. The hybrid density functionals are used as implemented. All structures were optimized without restrictions in gas phase under tight optimization conditions by using ultrafine grid in the computation of the two-electron integrals and their derivatives. The optimized structures were then characterized as true minima by vibrational frequency calculations. The solvent effect was then described by applying the polarizable continuum model (PCM) in its integral equation formalism variant (IEFPCM) [76]. In the case of MP2 normal optimization conditions were used. In the CCSD(T) [77] single point calculations the convergence on the energy was set to 10^−6^. For the purpose of benchmarking the ΔE values, representing the energy difference E**_K_**−E**_E_** in the corresponding solvent, are used.

The TautLYP was optimized using the general equation for exchange-correlation functional:

(1) $$E_{XC}=a.E_{X}^{HF}+b.E_{X}^{Slater}+c.\Delta E_{X}^{Becke}+d.E_{C}^{VWN}+e.\Delta E_{C}^{LYP}$$

where $\Delta E_{X}^{Becke}$ and $\Delta E_{C}^{LYP}$ are the generalized gradient approximations–the Becke’s 1988 exchange functional [57] and the correlation functional of Lee, Yang and Parr [62,78], respectively; $E_{C}^{VWN}$ is the VWN local-density approximation to the correlation functional [79].

Thus, $E_{XC}$ is governed by 5 parameters, which gives it a high extent of flexibility and possibility to be fitted against experimental data. In this case searching global minimum of the target function *S_T_* was performed according to the following equation:

(2) $$S_{T}=\frac{1}{n_{T}}\overset{n_{T}}{\underset{i=1}{\sum }}{\left(\frac{\Delta G_{i}^{exp}-\Delta E_{i}^{E_{XC}}}{\Delta G_{i}^{exp}}\right)}^{2}$$

where *n_T_* is the number of the tautomeric compounds included in the fitting (six compounds, Scheme 1), Δ*G^exp^* are the experimentally determined Gibb’s free energies 25 °C in cyclohexane (as described above) and $\Delta E^{E_{XC}}$ are the relative energies (difference between the energies of the keto and enol tautomeric forms of the same compound optimized in gas phase and calculated in cyclohexane as a single point) calculated with the functional defined by equation (1) at particular values of *a* − *e*. 

The optimization in respect to equation (2) was performed by using advanced simplex optimization of these optimization parameters (*m*, which means *m* + 1 dimensional optimization space and *m* vertices of the simplex). In this case *m* = 5. The method of Nelder and Mead was applied [80] for the optimization. The process was terminated when the standard deviation $\sigma^{2}$, defined by Equation (3), became less than 1 × 10^−9^.

(3) $$\sigma^{2}=\frac{\sum_{j=1}^{m+1}{\left(S_{T_{j}}-\bar{S_{T}}\right)}^{2}}{m+1}$$

where $S_{T_{j}}$ is the value of the optimization function (2), defined by a set of *a_j_* − *e_j_* parameters; $\bar{S_{T}}$ is the average value.

At that stage there are no substantial differences between the *a* − *e* values in the *m* vertices, because the simplex is too small.

In total, ten optimization procedures were performed until the end with a differing orientation of the initial simplex, yielding finally TautLYP with *a* = 0.4379; *b* = 0.5178; *c* = 0; *d* = 0.0910; *e* = 0.9174.

The TautLYP density functional can be used by the following Gaussian 09 input:

# BLYP IOp (3/76 = 1000004379,3/77 = 0000005178,3/78 = 0917400910).

## 4. Conclusions

TautData dataset containing precise information for the position of the tautomeric equilibrium of azodyes and Schiff bases was created. It contains information for 6 compounds in cyclohexane, 13–in carbon tetrachloride and 15–acetonitrile and can be used for benchmarking of density functional development.

Using the information for the position of the tautomeric equilibrium in cyclohexane of 6 core structures of industrially important azo- and azomethyne dyes, a new hybrid density functional, namely TautLYP, was fitted. Its reliability was checked by using the data from TautData, showing very good performance.

Benchmarking of existing DFT functionals was performed by using TautData and available structural information for some of the tautomers. The results have shown that MN12-SX, BHandH and M06-2X can be used to describe the tautomeric state of the core structures in the frame of ~0.5 kcal/mol error and correctly predict the tautomeric state in respect to a dominating tautomeric form. Among them, MN12-SX is the best performer, although it fails to describe the nonplanarity of some of the enol tautomers.

## Figures and Tables

**Table 1:** Experimental (ΔG_experimental_) and predicted relative stabilities (ΔE_predicted_) * of the tautomers of **1**–**6** in cyclohexane **.

Functional Name	ΔE_predicted_ kcal/mol						Linearity: ΔE_predicted_ = *a*. ΔG_experimental_ + *b*		
	1	2	3	4	5	6	*a*	*b*	R ***
TautLYP	1.49	0.39	0.22	1.54	0.80	−1.35	1.04	−0.01	0.996
MN12-SX [42]	2.00	0.48	−0.35	1.58	0.79	−0.60	0.91	0.25	0.936
BHandH [43]	1.01	0.22	−0.42	1.50	0.68	−2.15	1.22	−0.39	0.988
M06-2X [44]	1.88	0.82	0.18	2.23	1.41	−1.19	1.19	0.38	0.996
*HF* [30,45]	*1.16*	*0.28*	−*0.22*	*0.55*	−*0.14*	−*2.83*	*1.19*	−*0.71*	*0.906*
SOGGA11-X [46]	0.73	−0.65	−1.40	0.42	−0.42	−2.25	1.03	−1.04	0.975
M11 [47]	0.83	−0.89	−1.34	0.51	−0.13	−2.59	1.19	−1.11	0.986
BHandHLYP [43]	0.42	−0.65	−1.30	0.53	−0.25	−2.80	1.17	−1.18	0.996
CAM-B3LYP [48]	−0.44	−1.15	−1.71	0.31	−0.41	−3.58	1.27	−1.71	0.978
HISSbPBE [49]	−0.70	−1.03	−1.83	0.31	−0.64	−3.60	1.23	−1.78	0.965
ωB97X [50]	−0.15	−1.39	−1.88	0.07	−0.62	−3.59	1.28	−1.81	0.995
PBE0 [51]	−0.90	−1.08	−1.90	0.37	−0.54	−3.57	1.21	−1.79	0.951
PBEh1PBE [52]	−0.92	−1.19	−2.01	0.25	−0.65	−3.59	1.20	−1.87	0.958
APF [53]	−1.07	−1.19	−2.01	0.27	−0.63	−3.71	1.22	−1.91	0.944
ωB97X-D [54]	−0.60	−1.41	−2.00	0.08	−0.66	−3.81	1.29	−1.96	0.984
HSE06 [55]	−1.04	−1.20	−2.05	0.20	−0.72	−3.63	1.18	−1.92	0.951
LC-ωPBE [56]	−0.87	−1.41	−1.86	0.35	−0.34	−4.46	1.51	−2.08	0.950
APFD [53]	−1.45	−1.29	−2.11	0.26	−0.62	−4.07	1.28	−2.10	0.914
B3PW91 [57,58]	−1.34	−1.33	−2.17	0.14	−0.77	−3.91	1.22	−2.09	0.932
X3LYP [59]	−1.19	−1.49	−2.26	−0.16	−1.00	−3.80	1.14	−2.14	0.963
*MP2* [60]	*3.70*	*2.03*	*1.24*	*3.75*	*3.18*	*1.45*	*0.96*	*2.14*	*0.902*
B3LYP [61,62]	−1.36	−1.58	−2.37	−0.24	−1.09	−3.93	1.15	−2.26	0.957
TPSSh [63]	−1.61	−1.49	−2.35	−0.31	−1.22	−4.01	1.11	−2.31	0.930
τ-HCTHhyb [64]	−1.87	−1.58	−2.45	−0.12	−1.02	−4.31	1.22	−2.42	0.903
N12SX [42]	−1.95	−1.79	−2.58	−0.22	−1.16	−4.59	1.28	−2.60	0.913
TPSS [65]	−2.44	−1.73	−2.64	−0.68	−1.58	−4.58	1.08	−2.74	0.861
O3LYP [66,67]	−2.44	−2.06	−2.90	−0.48	−1.43	−4.85	1.25	−2.90	0.889
BMK [68]	−3.01	−3.16	−3.94	−0.74	−1.65	−5.99	1.57	−3.76	0.906
M06-HF [44]	6.34	4.92	4.55	4.00	3.81	2.72	0.70	4.09	0.609
***experiment*** ****	***1.35***	***0.42***	−***0.24***	***1.42***	***0.94***	−***1.31***			

* difference between the energies of the keto and enol tautomeric forms, positive value indicates more stable enol tautomer and vice versa; ** see Materials and methods for the computational details and experimental procedures; *** correlation coefficient; **** ΔG values (ΔG_experimental_), derived from the tautomeric constant in cyclohexane at room temperature, the corresponding molar fractions [in %] of the enol tautomer are 9, 33, 60, 8, 17, 90 for **1**–**6** correspondingly.

**Table 2:** Statistical parameters of the benchmarking (Table 1).

Functional Name	Type *	HF Exchange [%]	MAE **	MAPE *** [%]	MSE ****	MSPE ***** [%]
TautLYP	GH-GGA	43.79	0.08	8.4	0.01	84.6
MN12-SX	RSH-mGGA	25–0	0.31	31.5	0.16	1326
BHandH	GH-GGA	50	0.32	41.5	0.16	2336
M06-2X	GH-mGGA	54	0.46	71.5	0.25	8019
*HF*		100	0.64	58.1	0.72	5307
SOGGA11-X	GH-GGA	40.15	1.03	179	1.11	56,652
M11	RSH-mGGA	42.8–100	1.03	182	1.14	57,162
BHandHLYP	GH-GGA	50	1.11	179	1.26	50,795
CAM-B3LYP	RSH-GGA	19–65	1.59	254	2.61	100,203
HISSbPBE	RSH-GGA	0–60–0	1.68	265	2.97	109,721
ωB97X	RSH-GGA	15.77–100	1.69	278	2.95	124,477
PBE0	GH-GGA	25	1.70	271	3.08	118,265
PBEh1PBE	GH-GGA	25	1.78	287	3.34	133,408
APF	GH-GGA	23	1.82	290	3.53	134,506
ωB97X-D	RSH-GGA + D	22.2–100	1.83	296	3.48	139,486
HSE06	RSH-GGA	25–0	1.84	294	3.54	139,086
LC-ωPBE	RSH-GGA	0–100	1.86	289	3.94	127,711
APFD	GH-GGA + D	23	1.98	310	4.29	152,067
B3PW91	GH-GGA	20	1.99	316	4.23	159,313
X3LYP	GH-GGA	21.8	2.08	333	4.44	176,504
*MP2*		100	2.13	298	4.72	115,308
B3LYP	GH-GGA	20	2.19	352	4.95	196,692
TPSSh	GH-mGGA	10	2.26	353	5.31	193,046
τ-HCTHhyb	GH-mGGA	15	2.32	365	5.75	210,342
N12SX	RSH-GGA	25–0	2.48	391	6.52	239,972
TPSS	mGGA	0	2.71	411	7.72	253,502
O3LYP	GH-GGA	11.61	2.79	442	8.23	307,829
BMK	mGGA	42	3.51	586	13.1	582,436
M06-HF	GH-mGGA	100	3.96	709	16.5	930,805

* the following abbreviations are used in this column: +D, addition of dispersion corrections; GH, global hybrid; RSH, range-separated hybrid; GGA, generalized gradient approximation; mGGA, meta-GGA.;** Mean Absolute Error; *** Mean Absolute Percentage Error; **** Mean Square Error; ***** Mean Square Percentage Error.

**Table 3:** MAE and MAPE of the structural elements description of the tautomers of **1** and **6**.

Functional Name	MAPE [%]			MAE		Non-Planarity Description *
	Bonds	Angles	Total	Bonds [Å]	Angles [^o^]	
TautLYP	3.31	42.27	45.57	0.05	3.81	yes
MN12-SX	2.95	199.70	202.65	0.04	20.72	no
BHandH	4.95	199.99	204.93	0.07	20.75	no
M06-2X	2.61	114.56	117.17	0.04	12.26	yes
*HF*	4.53	48.04	52.57	0.06	4.34	yes
SOGGA11-X	2.76	140.41	143.16	0.04	14.91	no (**1**), yes (**6**)
M11	2.55	200.00	202.55	0.03	20.75	no
BHandHLYP	3.38	141.55	144.93	0.05	15.00	no (**1**), yes (**6**)

* according to the crystallographic data the Ph and naphthalene rings in the enol forms of **1** and **6** are twisted in respect of the N=N group (see Table S1 and Scheme S1 for more information).

**Table 4:** Experimentally determined ΔG values in kcal/mol units.

Cmpd	R	Solvent	
		CCl_4_	CH_3_CN
**1**	H		−0.03
**1.2**	OCH_3_		1.49
**2**	H	0.34	−0.15
**3**	H	−0.39	−0.47
**4**	H	1.40	0.30
**4.1**	N(CH_3_)_2_	1.33	0.37
**4.2**	OCH_3_	1.34	0.37
**4.3**	CH_3_	1.33	0.23
**4.4**	CN	1.42	0.55
**4.5**	NO_2_	1.48	0.47
**5**	H	0.79	−0.34
**5.1**	N(CH_3_)_2_	1.12	−0.45
**5.2**	OCH_3_	0.98	−0.20
**5.4**	CN	0.91	−0.10
**5.5**	NO_2_	1.00	−0.15

**Table 5:** Performance of the studied DFT functionals in respect of the data from Table 4.

Functional	MAE		MAPE [%]		MAPE [%] C_6_H_12_, CCl_4_, CH_3_CN
	CCl_4_	CH_3_CN	CCl_4_	CH_3_CN	
**TautLYP**	0.16	0.09	14.9	62.3	85.7
MN12-SX	0.18	0.18	15.8	97.3	144
BHandH	0.17	0.14	18.0	182	242
M06-2X	0.72	0.67	74.5	335	481
B3LYP	1.78	2.08	216	1401	1969

**Table 6:** Comparison between TautLYP and CCSD(T) in gas phase.

	ΔE_predicted_ kcal/mol						Linearity: ΔE_predicted_ = *a*. ΔG_experiment_ + *b*		
	1	2	3	4	5	6	*a*	*b*	R
TautLYP	2.49	0.80	0.09	2.34	1.57	−0.42	1.09	0.67	0.965
MP2	4.41	2.27	1.41	4.43	3.82	2.09	1.04	2.62	0.837
TautLYP *	2.70	0.63	−0.14	2.38	1.62	−0.21	1.10	0.69	0.922
MP3 *	4.03	2.64	2.11	4.56	4.00	1.11	1.24	2.54	0.976
MP4 *	1.67	0.41	−0.49	2.51	1.84	−0.48	1.10	0.44	0.909
CCSD(T) *	2.77	1.53	0.78	3.40	2.78	0.40	1.10	1.47	0.949

* using MP2 geometry.

## Supplementary Materials

The following are available online at https://www.mdpi.com/1420-3049/24/12/2252/s1, Scheme S1: Skeleton of **1** and **6**, Table S1: Experimental and predicted structural parameters of **1E**, **1K** and **6E**, Table S2: Predicted ΔE values in kcal/mol units.
