# Supplementary material for: Tautomerism in Azo and Azomethyne Dyes: When and If Theory Meets Experiment
Source: Molecules. 2019 Jun 17;24(12):2252. doi: 10.3390/molecules24122252 (PMC6631587; doi:10.3390/molecules24122252)
Supplement: Supplementary file 1 [file molecules-24-02252-s001.pdf]

molecules

Supporting Information

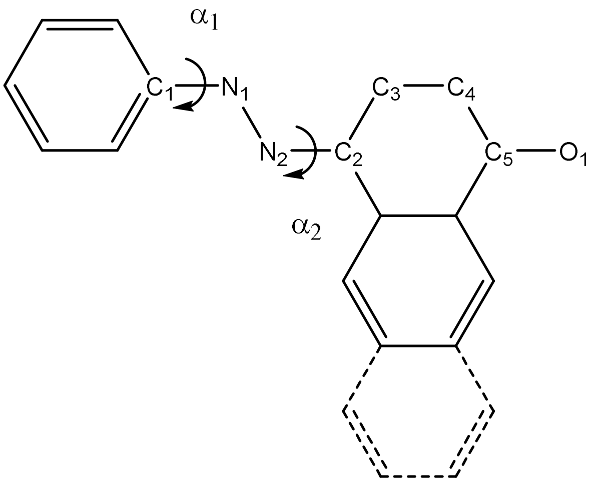

Scheme S1. Skeleton of **1** and **6**.

Table S1. Experimental and predicted structural parameters of **1E**, **1K** and **6E** (see Scheme S1 for the atom numbering).

	Bond lengths, Å							Dihedral angles, °	
	**C1-N1**	**N1-N2**	**N2-C2**	**C2-C3**	**C3-C4**	**C4-C5**	**C5-O1**	α_1	α_2
				**1E** (as OMe compound, CCDC 810701)					
exp	*1.429*	*1.266*	*1.429*	*1.372*	*1.401*	*1.376*	*1.370*	*9.0*	*11.5*
TautLYP	1.434	1.260	1.426	1.400	1.422	1.398	1.369	7.8	8.9
MN12-SX	1.412	1.258	1.405	1.385	1.399	1.382	1.353	0.0	0.0
BHandH	1.398	1.232	1.391	1.369	1.390	1.368	1.337	0.0	0.0
M06-2X	1.424	1.246	1.417	1.380	1.405	1.379	1.358	1.1	1.3
HF	1.421	1.219	1.418	1.364	1.407	1.364	1.348	8.6	10.5
SOGGA11-X	1.419	1.250	1.412	1.385	1.405	1.383	1.359	0.0	0.0
M11	1.427	1.243	1.420	1.376	1.407	1.376	1.360	0.0	0.0
BHandHLYP	1.412	1.238	1.406	1.374	1.398	1.372	1.351	0.0	0.0
				**1K** (as NMe compound, CCDC 810700)					
exp	*1.414*	*1.347*	*1.316*	*1.445*	*1.348*	*1.446*	*1.242*		
TautLYP	1.414	1.330	1.317	1.474	1.365	1.492	1.236		
MN12-SX	1.395	1.320	1.308	1.449	1.349	1.469	1.226		
BHandH	1.381	1.300	1.287	1.441	1.334	1.458	1.211		
M06-2X	1.400	1.319	1.301	1.458	1.345	1.477	1.222		
HF	1.401	1.337	1.268	1.473	1.328	1.481	1.200		
SOGGA11-X	1.399	1.319	1.305	1.457	1.349	1.475	1.226		
M11	1.403	1.319	1.297	1.463	1.342	1.480	1.223		
BHandHLYP	1.393	1.315	1.291	1.453	1.337	1.468	1.216		
				**6E** (as OMe compound, CCDC 820291)					
exp	1.454	1.251	1.419	1.369	1.408	1.362	1.361	6.8	14.2
TautLYP	1.434	1.260	1.426	1.393	1.432	1.391	1.368	9.6	13.2
MN12-SX	1.412	1.259	1.404	1.379	1.407	1.376	1.352	0.0	0.0
BHandH	1.398	1.232	1.391	1.362	1.399	1.361	1.336	0.0	0.0
M06-2X	1.424	1.246	1.416	1.373	1.415	1.371	1.357	5.8	8.7
HF	1.422	1.219	1.418	1.353	1.423	1.353	1.347	10.9	17.4
SOGGA11-X	1.418	1.251	1.412	1.378	1.414	1.376	1.358	4.8	6.8
M11	1.427	1.243	1.419	1.369	1.418	1.369	1.359	0.0	0.0
BHandHLYP	1.412	1.238	1.406	1.366	1.408	1.364	1.351	4.7	6.8

Table S2. Predicted ΔE values in kcal/mol units.

Cmpd	R	TautLYP		MN12-SX		BHandH		M06-2X		B3LYP	
		CCl_4	CH_3CN	CCl_4	CH_3CN	CCl_4	CH_3CN	CCl_4	CH_3CN	CCl_4	CH_3CN
**1**	H		-0.22		0.25		-0.69		0.44		-3.22
**1.2**	OCH_3		1.79		2.07		1.40		2.38		-1.49
**2**	H	0.34	-0.20	0.42	-0.17	0.17	-0.34	0.77	0.30	-1.64	-2.24
**3**	H	-0.26	-0.65	-0.39	-0.84	-0.46	-0.82	0.15	-0.21	-2.42	-2.87
**4**	H	1.44	0.24	1.47	0.18	1.40	0.23	2.13	1.00	-0.35	-1.59
**4.1**	$N(CH_3)_2$	1.46	0.24	1.59	0.30	1.48	0.33	2.22	1.08	-0.27	-1.51
**4.2**	OCH_3	1.62	0.39	1.75	0.45	1.63	0.46	2.39	1.24	-0.13	-1.37
**4.3**	CH_3	1.41	0.19	1.45	0.15	1.37	0.20	2.13	0.99	-0.39	-1.63
**4.4**	CN	1.85	0.58	1.95	0.61	1.76	0.54	2.50	1.32	0.07	-1.22
**4.5**	NO_2	1.80	0.55	1.85	0.52	1.66	0.46	2.44	1.27	-0.06	-1.33
**5**	H	0.70	-0.44	0.69	-0.55	0.59	-0.50	1.32	0.22	-1.19	-2.33
**5.1**	$N(CH_3)_2$	0.77	-0.41	0.93	-0.36	0.73	-0.38	1.45	0.33	-0.98	-2.18
**5.2**	OCH_3	0.91	-0.27	1.00	-0.28	0.85	-0.25	1.61	0.47	-0.91	-2.09
**5.4**	CN	1.07	-0.11	1.11	-0.15	0.92	-0.18	1.68	0.55	-0.84	-2.01
**5.5**	NO_2	0.98	-0.19	0.98	-0.28	0.80	-0.28	1.60	0.49	-0.99	-2.16
